# A New Food Source: Analyzing the Nutritional Value of Plant *Ferulago trachycarpa* Boiss

**DOI:** 10.1002/fsn3.71173

**Published:** 2025-11-09

**Authors:** Safinaz Elmasulu

**Affiliations:** ^1^ Department of Field Crops, Faculty of Agriculture Akdeniz University Antalya Türkiye

**Keywords:** edible plants, *Ferulago trachycarpa*, nutrition

## Abstract

The Umbelliferae family, consisting of annual or perennial herbs and rarely shrubs, takes up a place in our country's flora with 97 genera, and the *Ferulago* genus is represented by 28 species. *Ferulago trachycarpa* is a wild species traditionally used in human nutrition due to its sedative, digestive, carminative, and aphrodisiac properties. The purpose of this study was to determine the nutritional value of a plant sampled in Antalya Province, Alanya District, Gökbel Plateau during the pre‐flowering period. The microelement, energy, carbohydrate, fat, total ash, water, and crude cellulose values were investigated. Evaluating the results, the energy value for a 100‐g fresh sample was 73.51 kcal, the water content was 68.35%, the ash amount was 6.79%, the protein amount was 2.59%, the fat amount was 0.71%, the carbohydrate amount was 14.19%, and the fiber amount was 7.37%. When looking at the contents of the microelements, iron was detected at 1.85 mg/100 g, phosphorus at 28.69 mg/100 g, calcium at 2031.96 mg/100 g, magnesium at 68.57 mg/100 g, and potassium at 140.06 mg/100 g, but sodium and zinc were not detected. The amount of total ash suggests a mineral composition comprising potassium, calcium, and iron as the main elements. The present study inferred that *Ferulago trachycarpa* would serve as a beneficial source of protein and energy, as well as micronutrients in the form of a leafy vegetable for human consumption. Therefore, agricultural domestication for the commercial production of the *Ferulago trachycarpa* species needs to be investigated to serve commercial production as a functional food.

## Graphical abstract

*Ferulago trachycarpa*, the subject of this study, is one of the many plants found in our country's flora and used in nutrition. It is an important part of both human and animal nutrition, especially in areas where food resources are limited, as it spreads in habitats above 1000 m. This study aimed to determine and introduce the nutritional value of the species, which has many ethnobotanical uses, and the results obtained showed that the species is rich in calcium, potassium, iron, and total ash.

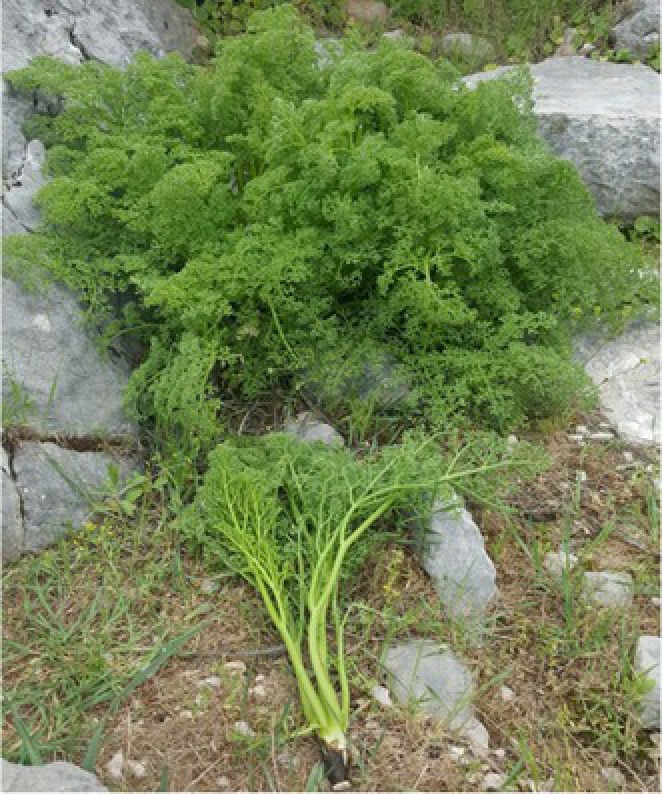

A figure of *Ferulago trachycarpa* in its natural habitat.

## 1 Introduction

Plants, both in the world and in Türkiye, are used in human nutrition by being culturally produced or collected from nature in wild form. Many plants in the flora of Türkiye are used as fresh, cooked, or flavoring in the early development periods in the geographies where they spread. Thanks to our rich biodiversity, the number of these plants included in the human diet is quite high. The rising interest in natural products in recent years means these species are also important in terms of being a source of livelihood, especially for rural residents, as they are a subject of trade in local markets (Lachat et al. 2018; Hunter et al. 2019; Vermeulen et al. 2020; Belgacem et al. 2021; Medeiros et al. 2022; Lawrence 2024).

The many wild plant species that are well‐known and consumed by indigenous people in the regions where they are distributed are also conveyed to the local markets and offered for the consumption of different social groups. Besides the nutritional benefits of these plants, which are cooked as vegetables or used fresh in salads, many of their pharmacological properties also make them important and valuable.

The largest group of these plants is spices, which are used as an indispensable part of food all over the world. In addition to their flavoring properties, spices are also recognized for their various health benefits, which contribute significantly to their extensive usage (Asaduzzaman and Toshiki 2018; Rahmatullah et al. 2010).

Many species in nature are used for nutrition by local communities, but they are agricultural genetic resources that have not been commercialized or evaluated in terms of research and development. In terms of these plants, which are defined as neglected and underutilized species, our natural environment is very rich (Hunter et al. 2019; Joshi et al. 2019).

In comparison to commonly consumed and traded foods, the proportion of edible weeds in the human diet is low. However, these plants are important as a source of livelihood, as well as for daily diets in geographies where food resources are scarce. The fact that edible grasses provide an alternative source for many macro‐ and microelements, as well as adding dietary diversity, should not be overlooked.

In edible herbs, which are generally consumed for a limited period of time during the early vegetation period, the number of macro‐ and micro‐elements and their amounts in the plant vary depending on environmental factors. For this reason, the nutritional values of herbs collected from nature and consumed are not standard, unlike commercial products, and vary throughout the year depending on many factors, especially the climate.

It is also worth noting that these neglected plants may have nutritional contents similar to those of commercial products. It would be reasonable to think that neglected biological resources will be an important alternative for possible nutritional problems that may arise with rapid population growth or environmental changes such as global warming.

Edible wild plants have been consumed extensively in our country for years, especially in rural areas. Recently, with the increase in people's interest in natural products, the demand for edible weeds has also increased. Reasons such as society's growing awareness of the importance of the natural environment and consumers' starting to change their behavior accordingly, an increase in food‐ and nutrition‐related health problems, and society's growing awareness with the motto “Let your food be your medicine” form the dynamics of this fact. There are 51 taxa belonging to the genus *Ferulago* in the Apiaceae family, which is represented by 97 genera in the flora of our country (Guner et al. 2012) and is distributed in Europe, Asia, and Africa (Pimenov and Leonov 1993). *F. trachycarpa*, an element of the Eastern Mediterranean phytogeographic area, is distributed in the Eastern Aegean Islands, Syria, and Lebanon. The species has a perennial, tall, and glaucous color, with an angled, sulcate stem up to 125 cm in length and a panicled thyrse inflorescence. The plant, which blooms in June–August, spreads naturally on rocky slopes at an altitude of 870–2900 m in the Western, Southwestern, and Southern Anatolian regions of Türkiye (Akalın and Koçyigit 2010; Peşmen 1972).

*Ferulago* species have been historically utilized in traditional medicine as sedatives, tonics, digestives, carminatives, aphrodisiacs, and for the treatment of intestinal worms and hemorrhoids. The fresh base leaves of the plant are collected from nature in the spring and consumed as a salad. In some regions, the mature seeds of the plant are also used as a spice after being dried and ground (Akalın and Koçyigit 2010).

In recent studies, it has been determined that Ferulago species are effective for cytotoxic, antitumor, and memory functions (Heidari et al. 2014; Hritcu et al. 2015; Mirzapour et al. 2015; Shahneh et al. 2013).

Furthermore, it is employed as a source of nourishment in brine and as a means of enhancing taste and extending shelf life in dairy items, notably cheese (Satir 2006). The decoctions prepared from the roots and leaves of the *F. trachycarpa* plant have been reported to be used as aphrodisiacs and in stomach disorders (Bulut et al. 2014).

Previous studies have focused on the essential oil composition and pharmacological assessment of the *F. trachycarpa* plant (Akalın and Koçyigit 2010; Ay et al. 2017; Borelli et al. 2022; Bulut et al. 2014; Erdemoglu et al. 2008; Garcia‐Herrera et al. 2014; Heidari et al. 2014; Hritcu et al. 2015; Karakaya et al. 2019; Mirzapour et al. 2015; Ozhatay and Akalin 1998; Satir 2006). However, no studies have been identified that disclose the nutritional content of this species.

In this study, the nutritional values of the fresh consumption of the *F. trachycarpa* plant, whose many organs, from root to seed, are used in human nutrition and traditional medicine prescriptions for therapeutic purposes in the geographies where it spreads, were investigated and aimed to reveal the importance of the plant as an alternative vegetable in human nutrition (Ozhatay and Akalin 1998).

## 2 Materials and Methods

### 2.1 Material

In the study, the above‐ground parts of the *F. trachycarpa* plant sampled from Antalya Province, Alanya District, and Gökbel Plateau were used as plant material. The topography is dominated by stony and occasionally steppe vegetation formed by limestone stony scree. Located at the transition between the Mediterranean and Irano‐Turanian Phytogeographic Regions, the region is also subject to seasonal stream flows. The species' distribution points in the region are not steppe areas but rather stony slopes at altitudes of 1600–1700 m, in the vicinity of seasonal water flows where moisture balance is maintained. It has been determined that the species does not prefer relatively deep soil areas; it prefers limestone rocky slopes with stony scree.

Varieties developed for cultural production are genetically stabilized and give standard crops under field or greenhouse conditions. Unlike cultivated varieties, plants in nature are directly affected by changing environmental conditions. In order to survive and ensure the continuation of its generation, the plant takes measures under the stress conditions it encounters, and this causes it to synthesize different metabolites. In the stress conditions, the amounts of nutrients and minerals contained in edible herbs collected from nature may vary from year to year, interannually, and seasonally, as well as between locations.

Consequently, the data obtained by comprehensively sampling from one location in the study were evaluated comparatively with the Türkiye averages in the National Food Composition Database (TURKOMP) of species with similar consumption and the averages of our species, not by years or locations.

It was taken as a sample during the vegetative period, when the plant is generally consumed and transported to the laboratory fresh for analysis. The plant material employed in the study was gathered from the wild and does not undergo any form of cultivation. To accurately represent the population, bulk samples were meticulously prepared utilizing genotypes from similar developmental stages. The samples were maintained at low temperatures in ice cassettes and subsequently transported to the laboratory for detailed analysis.

### 2.2 Method

In the study, NMK 161 was used to analyze microelements in *F. trachycarpa* samples; Turkish Food Codex Food Labeling and Consumer Information Regulation (RG: 26.01.2017) was used as an energy and carbohydrate analysis method; AGAC 2011‐992.23 was used to analyze protein; and TS EN ISO 659 was used as an oil analysis method; TS 4888 was used to analyze total ash; TS 1129 ISO 1026 was used to determine moisture/water content; and TS 6932, April 1989, was used for the determination of crude fiber content. All analytical measurements were performed in duplicate to ensure methodological rigor and reproducibility.

*Ash Determination*: A 5 g sample was subjected to incineration in a muffle furnace at 525°C ± 10°C to determine ash content, a measure of mineral and salt content. The percentage ash was calculated from the weight of the resulting gray–white residue.

Moisture/Water Determination for the determination of moisture/water by drying under low pressure, a homogenized sample of 5 g was weighed and dried in a vacuum oven at 70°C until the sample reached a constant weight, then weighed, and the percentage of moisture/water was calculated.

*Protein determination*: In the method based on the principle of measuring the nitrogen gas released as a result of dry combustion of the sample with a suitable detector, 100–200 mg of the ground sample was added to the apparatus, two parallel readings were made, and the % protein was calculated using the average of the values obtained.

Cellulose Determination A 1 g sample was boiled successively with sulfuric acid and potassium hydroxide solutions, and the residue was filtered on a glass filter, separated, washed, dried, weighed, and burned at 550°C ± 25°C. The amount of crude cellulose in the sample was determined by calculating the weight loss after incineration.

Oil Determination A 5 g sample was transferred to an extraction cartridge, covered with oil‐free hydrophilic cotton, and extracted with petroleum ether for 6 h in a cartridge extractor. The solvent was then distilled, dried in an oven, cooled in a desiccator, and weighed to calculate % oil.

Microelement Analysis In the study utilizing the method “NMKL‐161‐1998 Metals Determination by Atomic Absorption Spectrophotometry After Wet Digestion in A Microwave Oven,” 0.5–1.0 g dry matter equivalent of the homogenized samples was transferred to microwave containers, HNO3 and H2 O2 were added to it, and subsequently, the containers were subjected to combustion in the microwave under a suitable program. Pure water was added to the cooled containers, and the amounts of the elements were determined by reading after the volume was adjusted accordingly.

*Carbohydrate Amount*: The carbohydrate content of the sample was estimated by difference, calculated as: Carbohydrates (%) = Dry matter (%) – [Protein (%) + Fat (%) + Fiber (%) + Ash (%)].

*Energy*: Protein, fat, and carbohydrate values were determined via a calculation that involved multiplying and summing their respective values by factors specified within the Food Labeling and Consumer Information Regulation.

## 3 Results and Discussion

Numerous studies have been conducted on edible herbs in many different places in the world and in Türkiye. These studies have been conducted in a variety of areas, including the use of wild herbs, ethnobotanical uses, nutritional content, essential oil content, fixed oil values of seeds, phytochemical content of the plant, or the significance of agriculture as a source of income for rural residents (Ay et al. 2017; Baser 2002; Bharucha and Pretty 2010; Borelli et al. 2022; Cinar et al. 2017; Demir et al. 2020; Garcia‐Herrera et al. 2014; Glew et al. 2005; Hailu and Addis 2016; Medak and Singha 2017; Tuncturk et al. 2017; Xu et al. 2020; Yildirim et al. 2001; Yucel et al. 2012). The fact that *Ferulago* species are consumed for different purposes in our country has attracted the attention of many researchers working in related fields (Baser 2002; Dikpınar et al. 2018; Erdemoglu et al. 2008; Karakaya et al. 2019).

The energy, carbohydrate, fat, total ash, water, and crude fiber values obtained as a result of the analyses carried out in this study to reveal the nutritional value of the *F. trachycarpa* plant are shown in Table 1, and the results of the mineral analysis for determination and quantification are summarized in Table 2, along with their limits. Türkiye's average nutritional and mineral substance values of some natural edible vegetables collected from nature and consumed fresh in our country (Anonymous 2014) are presented in Table 3 together with the results we obtained in the study.

**TABLE 1:** Energy, carbohydrate, protein, lipid, total ash, water, and crude fiber values.

Analysis	Result	Analysis method
Energy	73,51 kcal/100 g	Turkish Food Codex Regulation on Food Labeling and Consumer Information (RG: 26.01.2017)
Carbohydrate	14.19%	
Crude protein	2.59%	AGAC 2011–992.23
Crude fat	0.71%	TS EN ISO 659
Total ash	6.79% (m/m)	TS 4888
Water	68.35% (m/m)	TS 1129 ISO 1026
Crude fiber	7.37%	TS 6932 April 1989

*Note:* The component values are for an edible 100 g of food. Measurement uncertainty does not include sampling and is calculated using *k* = 2 at a 95% confidence interval.

**TABLE 2:** Results of mineral analysis in the *F. trachycarpa* plant.

Microelement	Result (mg/100 g)	Analysis method	LOD/LOQ
Copper (Cu)	0.1337	NMKL 161	‐/0.800
Zinc (Zn)	—		‐/5.800
Iron (Fe)	1.8536		‐/8.500
Phosphorus (P)	28.6865		‐/25.900
Calcium (Ca)	2031.9623		‐/21.500
Magnesium (Mg)	68.5731		‐/9.100
Sodium (Na)	—		‐/17.900
Potassium (K)	140.0632		‐/24.600

*Note:* LOD: detection limit, LOQ: measurement limit. Measurement uncertainty does not include sampling and is calculated using *k* = 2 at a 95% confidence interval.

**TABLE 3:** Comparison of some commonly consumed fresh vegetables and the *F. trachycarpa* plant in terms of nutritional values.

	*Ferulago trachycarpa* Boiss.	Lambs quarters *Chenopodium album* L.	Glassworth *Salicornia europaea* L.	Common Mallow *Malva silvestris* L.	Chicory *Cichorium intybus* L.	Curly dock *Rumex crispus* L.	Indian knotgrass polygonum cognatum meissn.	Rock samphire crithmum maritimum L.	Watercress *Nasturtium officinale* R.Br., Aiton.	Crown daisy *glebionis coronaria* (l.) spach, syn: *Chrysanthemum segetum* L.	*Gundelia tournefortii* L.	Salsify *scorzonera cana* (C.A.Mey.) griseb.	Alexanders *Smyrnium olusatrum* L.
Energy (kcal)	73.51	20	36	49	22	25	60	26	33	24	26	39	28
Water (%)	68.35	92.64	87.18	84.94	91.08	90.55	77.94	88.87	87.67	90.26	90.44	87.6	89.16
Ash (%)	6.79	1.57	3.02	2.01	1.54	1.13	3.05	2.25	2.33	1.3	1.46	1.8	0.66
Protein (%)	2.59	0.98	0.86	2.94	0.53	1.85	2.06	0.31	4.85	1.58	1.15	0.19	0.11
Fat (%)	0.71	0.35	0.49	0.57	0.28	0.36	0.65	0.39	0.4	0.31	0.13	0.83	0.12
Carbohydrate (%)	14.19	2.25	5.82	6.6	2.12	1.06	6.78	2.48	0.33	0.84	3.45	6.03	3.37
Fiber (%)	7.37	2.21	2.63	2.95	4.45	5.05	9.52	5.7	4.42	5.71	3.37	3.55	5.17
Iron (mg)	1.85	1.01	2.11	10.81	10.5	4.92	37.43	2.29	23.17	8.73	3.96	10.6	3.7
Phosphorus (mg)	28.69	52	24	67	31	54	43	22	41	46	22	29	46
Calcium (mg)	2031.96	69	117	267	138	76	275	225	197	239	191	233	180
Magnesium (mg)	68.57	76	121	60	33	34	78	46	26	53	19	54	38
Potassium (mg)	140.06	427	193	605	461	395	448	313	406	555	519	352	593
Sodium (mg)	—	237	1064	57	47	37	2	368	104	198	8	2	3
Zinc (mg)	—	0.41	0.23	0.82	0.39	0.43	0.71	0.26	0.28	1.97	0.3	0.32	0.25

*Note:* The component values are for an edible 100 g of food. The values given in the table, which belong to some commonly consumed fresh vegetables, are taken from the National Food Composition Database (TURKOMP 2025).

In the study, a total of eight minerals, including copper, zinc, iron, phosphorus, calcium, magnesium, sodium, and potassium, were investigated, but sodium and zinc were not detected. When the values of mineral analyses were examined, calcium drew attention among the plant values. The *F. trachycarpa* plant, which has a considerably higher value than Türkiye's average of freshly consumed edible herbs, is compared in Table 3 and is thought to have the potential to serve as a valuable alternative vegetable in diets as a calcium source. In terms of other minerals, the total ash content of the plant, which is thought to possess the potential to aid diets, also supports this situation. The amount of ash gives information about the amount of inorganic matter contained in the plant and, therefore, the amount of mineral matter in inorganic form. Particularly, it becomes crucial when it is not possible to analyze all macro‐ and microelements. In this context, it is noteworthy that the ash amount values obtained from *F. trachycarpa* are considerably higher than the Türkiye average values of the vegetables in Table 3.

When the *F. trachycarpa* plant is compared with the Türkiye averages of the plants in Table 3 in terms of the amount of energy obtained, the *F. trachycarpa* plant was seen to be higher than “Madımak” (*Polygonum cognatum* Meissn.), “Ebegümeci” (*Malva silvestris*), and “Tekesakalı” (*Scorzonera cana* (C.A. Mey.) Griseb) plants that are commonly collected from nature and consumed as fresh vegetables.

The protein amount of the *F. trachycarpa* plant was seen to be lower than that of “Su Teresi” ( *Nasturtium officinale* R.Br., Aiton), but close to that of “Ebegümeci” (*Malva silvestris*), and higher than other edible wild plants compared.

The amount of water contained in the plant affects its taste and texture properties, and also plays an important role in the fresh consumption of the plant. Despite the fact that the amount of water in *F. trachycarpa* was comparatively lower than that of the species listed in Table 3, it was seen to be at an acceptable level. This situation was supposed to have been caused by either harvest time or environmental factors.

The oil content value of the *F. trachycarpa* plant has the highest value after “Tekesakalı” (*Scorzonera cana* (C.A. Mey.) Griseb) among the species in Table 3.

In relation to the quantity of carbohydrates, the *F. trachycarpa* plant exhibited a significantly greater value compared to the mean of the other edible plants, as presented in Table 3.

The *F. trachycarpa* plant was found to have a higher fiber content value when compared to the average of other edible wild species listed in Table 3, except for the “Madımak” (*Polygonum cognatum* Meissn.) species.

The quantity of fiber present in a plant is directly correlated with its developmental stage (Borelli et al. 2022; Yildirim et al. 2001), In their study, they reported that the *F. trachycarpa* plant, which they sampled from the Mediterranean Region, had the highest fiber ratio of 10.80 g/100 g among the 41 species they investigated in three regions in Türkiye. Despite the fact that the crude fiber value derived from the study (7.37%) was near this value, differences were attributed to the fact that the plants were collected at various developmental stages.

## 4 Conclusion

A significant proportion of global food consumption is derived from a limited range of plant and animal species, with 12 plant and 5 animal species accounting for 75% of this consumption. Additionally, the consumption of three primary products, namely wheat, corn, and rice, accounts for over 50% of the total calorie intake. With the ever‐increasing human population, resources regarding nutrition are becoming more and more significant. However, people's quest for differences in the foods they consume daily is becoming a phenomenon that cannot be disregarded. When all these factors are considered together, the significance of plants that are consumed within our local region is also underscored in terms of genetic resources. Edible plants, which are consumed extensively, particularly in regions with a high level of biodiversity, are also an essential source of income for rural regions.

In this study, the nutritional value of the *F. trachycarpa* species, which is used as a therapeutic in our country as well as consumed as a vegetable in the early vegetation period, was investigated, and the results obtained were evaluated.

The young shoots of the species are consumed as vegetables in the places where it is naturally distributed, and it attracts attention with its aromatic odor.

Although many studies have been conducted and published on the amount and content of essential oil in the species *Ferulago trachycarpa* Boiss., no study has been found investigating its nutritional value.

Consequently, this study makes a significant contribution to understanding the nutritional value of this species.

In nutrition, the ash content of vegetables is often an important parameter for determining the mineral content of a vegetable. “Ash” refers to the amount of incombustible, inorganic material left behind when food is burnt at high temperatures. These substances are usually minerals: calcium, potassium, magnesium, iron, phosphorus, and sodium. The ash content of vegetables provides an indication of their total mineral content. This measurement is used to determine the vegetable's contribution to vital functions such as bone health (calcium and magnesium), muscle function (potassium), blood production (iron), and acid–base balance (sodium and potassium) (Gibson et al. 2010; Kibar and Temel 2016; Zvěřina et al. 2025).

The high amount of ash in the *F. trachycarpa* plant, which is particularly notable for its high calcium content, also increases its nutritional value in terms of total mineral matter. When compared to the nutritional values of other edible botanicals, it was determined that the values obtained in our study were generally high.

In light of these results, it was concluded that the *F. trachycarpa* plant is an important natural plant species for diversifying and enriching human nutrition. For agricultural production, it is important to expand this preliminary study by sampling the plant from different locations, investigating its suitability for cultural production, determining appropriate propagation and cultivation conditions, and then initiating breeding studies to investigate its potential for introducing it to the functional food market.

## Author Contributions

Safinaz Elmasulu: All study for the article was carried out by the corresponding author.

## Ethics Statement

The author has nothing to report.

## Conflicts of Interest

The author declares no conflicts of interest.

## Data Availability

The data that support the findings of this study are available from the corresponding author upon reasonable request.
